# A novel prognostic signature based on immune-related genes of diffuse large B-cell lymphoma

**DOI:** 10.18632/aging.203587

**Published:** 2021-10-05

**Authors:** Zizheng Wu, Qingpei Guan, Xue Han, Xianming Liu, Lanfang Li, Lihua Qiu, Zhengzi Qian, Shiyong Zhou, Xianhuo Wang, Huilai Zhang

**Affiliations:** 1 Departments of Lymphoma, Tianjin Medical University Cancer Institute and Hospital, National Clinical Research Center of Cancer, Key Laboratory of Cancer Prevention and Therapy, Tianjin’s Clinical Research Center for Cancer, Sino-US Center for Lymphoma and Leukemia Research, Tianjin 300060, China

**Keywords:** diffuse large B-cell lymphoma, immune-related genes, overall survival, gene expression omnibus database, prognostic signature

## Abstract

Diffuse large B-cell lymphoma (DLBCL) presents a great clinical challenge and has a poor prognosis, with immune-related genes playing a crucial role. We aimed to develop an immune-related prognostic signature for improving prognosis prediction in DLBCL.

Samples from the GSE31312 dataset were randomly allocated to discovery and internal validation cohorts. Univariate Cox, random forest, LASSO regression and multivariate Cox analyses were utilized to develop a prognostic signature, which was verified in the internal validation cohort, entire validation cohort and external validation cohort (GSE10846). The tumor microenvironment was investigated using the CIBERSORT and ESTIMATE tools. Gene set enrichment analysis (GSEA) was further applied to analyze the entire GSE31312 cohort. We identified four immune-related genes (CD48, IL1RL, PSDM3, RXFP3) significantly associated with overall survival. Based on discovery and validation cohort analyses, this four-gene signature could classify patients into high- and low-risk groups, with significantly different prognoses. Activated memory CD4 T cells and activated dendritic cells were significantly decreased in the high-risk group, and these patients had lower immune scores. GSEA revealed enrichment of signaling pathways, such as T cell receptor, antigen receptor-mediated, antigen processing and presentation of peptide antigen via MHC class I, in the low-risk group. In conclusion, a robust signature based on four immune-related genes was successfully constructed for predicting prognosis in DLBCL patients.

## INTRODUCTION

Diffuse large B-cell lymphoma (DLBCL), the most frequent pathologic subtype of malignant lymphoma, poses challenges for classification and treatment [1]. Moreover, evidence from clinical and biological studies has indicated that DLBCL is an aggressive severe and complicated disease with a broad spectrum of genetic, phenotypic and clinical heterogeneities [2]. Despite a vast improvement in the survival rate (50% ~ 60%), the heterogeneous nature of this disease elicits different survival outcomes for DLBCL patients undergoing routine treatment (rituximab, cyclophosphamide, doxorubicin, vincristine, prednisone (R-CHOP)) [3]. In general, finding novel anti-DLBCL pharmaceutical targets, either alone or in combination with R-CHOP therapy, is crucial for survival enhancement or alternative measures for ineligible, relapsed or refractory cases [4].

The international prognostic index (IPI), which includes age, tumor stage, Eastern Cooperative Oncology Group (ECOG) performance, number of extranodal sites and lactate dehydrogenase (LDH) level, is widely employed for the clinical evaluation of DLBCL patient prognosis [5]. However, IPI does not consider the molecular heterogeneity of DLBCL, and the marked differences in patient survival, even among patients with similar or the same IPI [6]. Current DLBCL studies focus on recognizing novel risk stratification and prognostic biomarkers to predict survival outcomes and treatment response or to identify patients eligible for more aggressive therapies. At the same time, prognostic biomarkers may shed light on current and future potential therapies.

With advances in human gene sequencing technology, increasing attention has been given to gene-based biomarkers [7]. Furthermore, there is growing evidence that immune-related genes and the tumor immune microenvironment (TME) are crucial for malignant tumor progression and response to therapy [8, 9]. Therefore, an immune-related gene signature that enables physicians to estimate DLBCL prognosis and characterize the TME in these patients is urgently needed.

Here, we developed a reliable prognostic signature (PS) for DLBCL using immune-related genes and validated the clinical feasibility of this signature in DLBCL patients. Immune cell infiltration and the TME of patients with different risk scores are comprehensively described.

## RESULTS

### Prognostic genes recognition and PS construction

**Figure 1:**
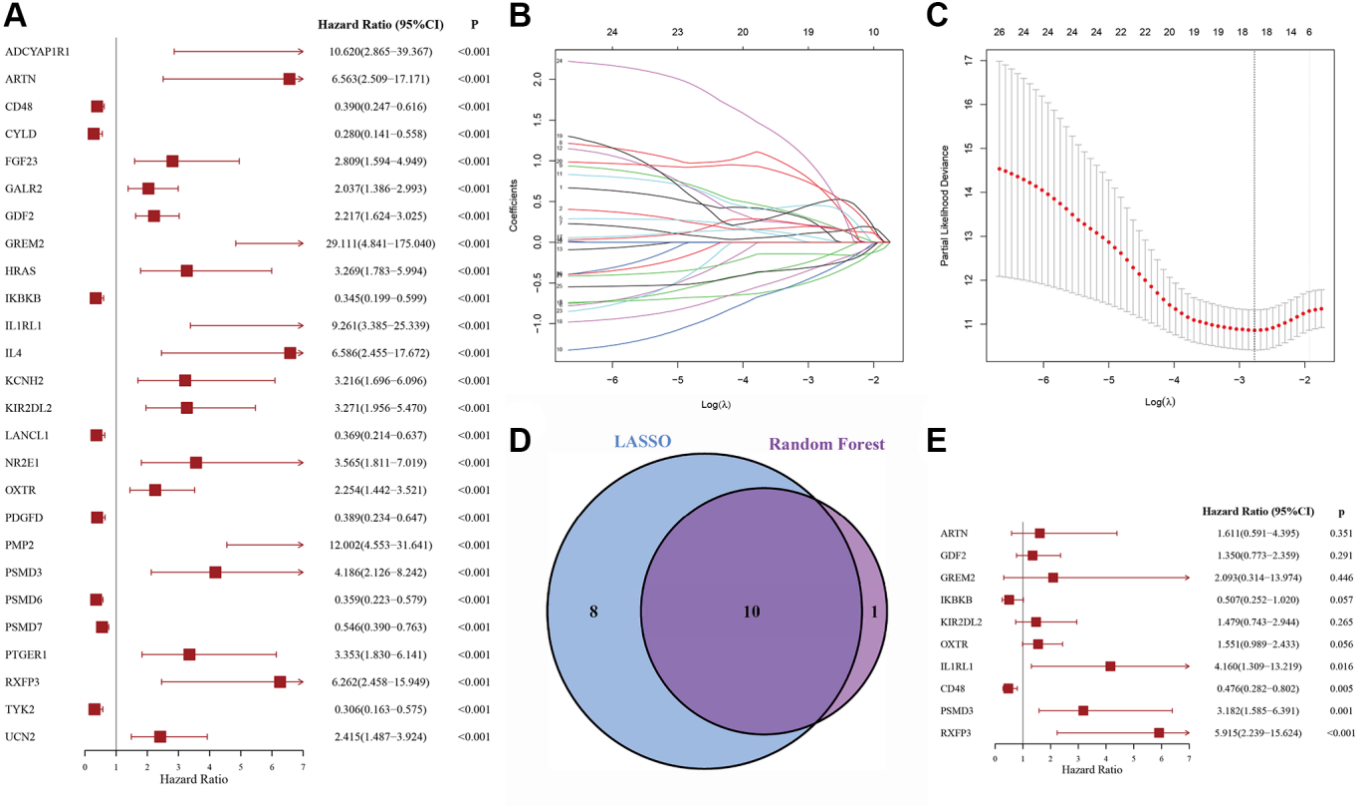
**The process of variable selection.** (**A**) Forest map of 26 candidate immune-related genes selected by univariate Cox regression analysis associated with DLBCL overall survival in the discovery cohort. (**B**, **C**) The performance of least absolute shrinkage and selection operator (LASSO) analysis. (**D**) LASSO and random forest analysis intersecting genes were selected. (**E**) Forest map of multivariate Cox regression analysis to establish a prognostic signature.

Overall, 426 DLBCL patients from the GSE31312 dataset were arbitrarily placed into a discovery cohort (DC, *n* = 213) or an internal validation cohort (IVC, *n* = 213). We then conducted univariate Cox regression analysis on the immune-related genes expression profiles in the DC and 26 candidate genes were significantly correlated with overall survival (OS) (*p* < 0.001) (Figure 1A). To retrieve the most significant genes with predictive abilities, random forest and LASSO regression analyses were performed synchronously to reduce the volume and select the most relevant genes (Figure 1B, 1C). Eleven and eighteen genes were chosen, respectively; ten genes intersected and were deemed to have the greatest predictive power for OS (Figure 1D). Multivariable Cox regression was then carried out (Figure 1E), which revealed four risk genes: *CD48* (HR = 0.476, 95% CI: 0.282–0.802, *p* = 0.005), *IL1RL1* (HR = 4.160, 95% CI: 1.309–13.219, *p* = 0.016), *PSMD3* (HR = 3.182, 95% CI: 1.585–6.391, *p* = 0.001), and *RXFP3* (HR = 5.915, 95% CI: 2.239–15.624, *p* < 0.001).

Furthermore, a risk score (RS) system was established, according to the levels of these four genes and the corresponding coefficient obtained from multivariable Cox regression analysis. The formula was as follows: RS = (−0.794 × *CD48* levels) + (2.243 × *IL1RL1* levels) + (1.440 × *PSDM3* levels) + (1.348 × *RXFP3* levels). We then computed the RS for each patient and set the median as the cutoff to divide them into high-risk (HR) and low-risk (LR) groups.

**Figure 2:**
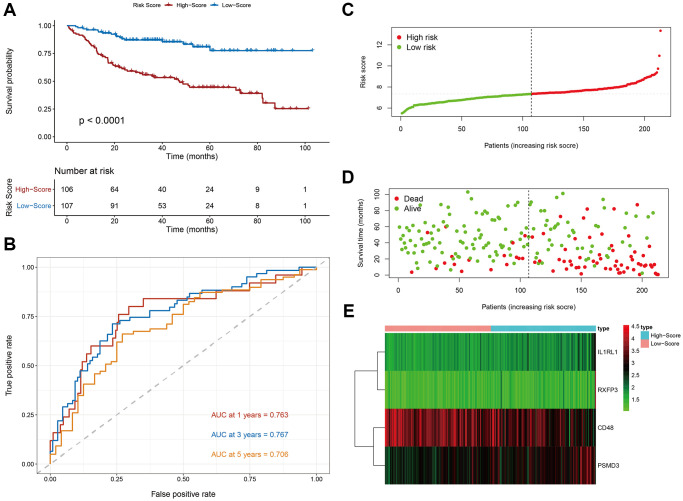
**Evaluation of the prognostic signature in the discovery cohort.** (**A**) Kaplan-Meier plots of overall survival between high- and low-risk patients. (**B**) Time-dependent receiver operating characteristic (ROC) curve analysis. (**C**) The risk score distribution. (**D**) The survival status distribution. (**E**) Expression heatmap of the four immune-related risk genes.

As shown in Figure 2A, Kaplan-Meier survival analysis of the DC showed that HR patients exhibited significantly worse prognosis, compared to LR patients (log-rank *p* < 0.001). The OS rates at 3 and 5 years for the HR patients were 69.33% and 54.54%, respectively, while the corresponding rates for the LR patients were 85.44% and 83.31%, respectively. The PS area under the curve (AUC) analysis demonstrated its excellent accuracy in estimating DLBCL patient OS (1-year AUC = 0.763, 3-year AUC = 0.767, 5-year AUC = 0.706) (Figure 2B).

We next plotted risk curves and survival status scatter plots to illustrate the RS and OS of each DLBCL patient in the DC, and a worse prognosis was significantly associated with a higher RS (Figure 2C, 2D). Moreover, using a heatmap, we demonstrated the HR and LR gene expression profiles of both groups (Figure 2E). We revealed that *CD48* was significantly upregulated in LR patients and that *IL1RL*, *PSDM3*, and *RXFP3* were strongly elevated in HR patients.

**Figure 3:**
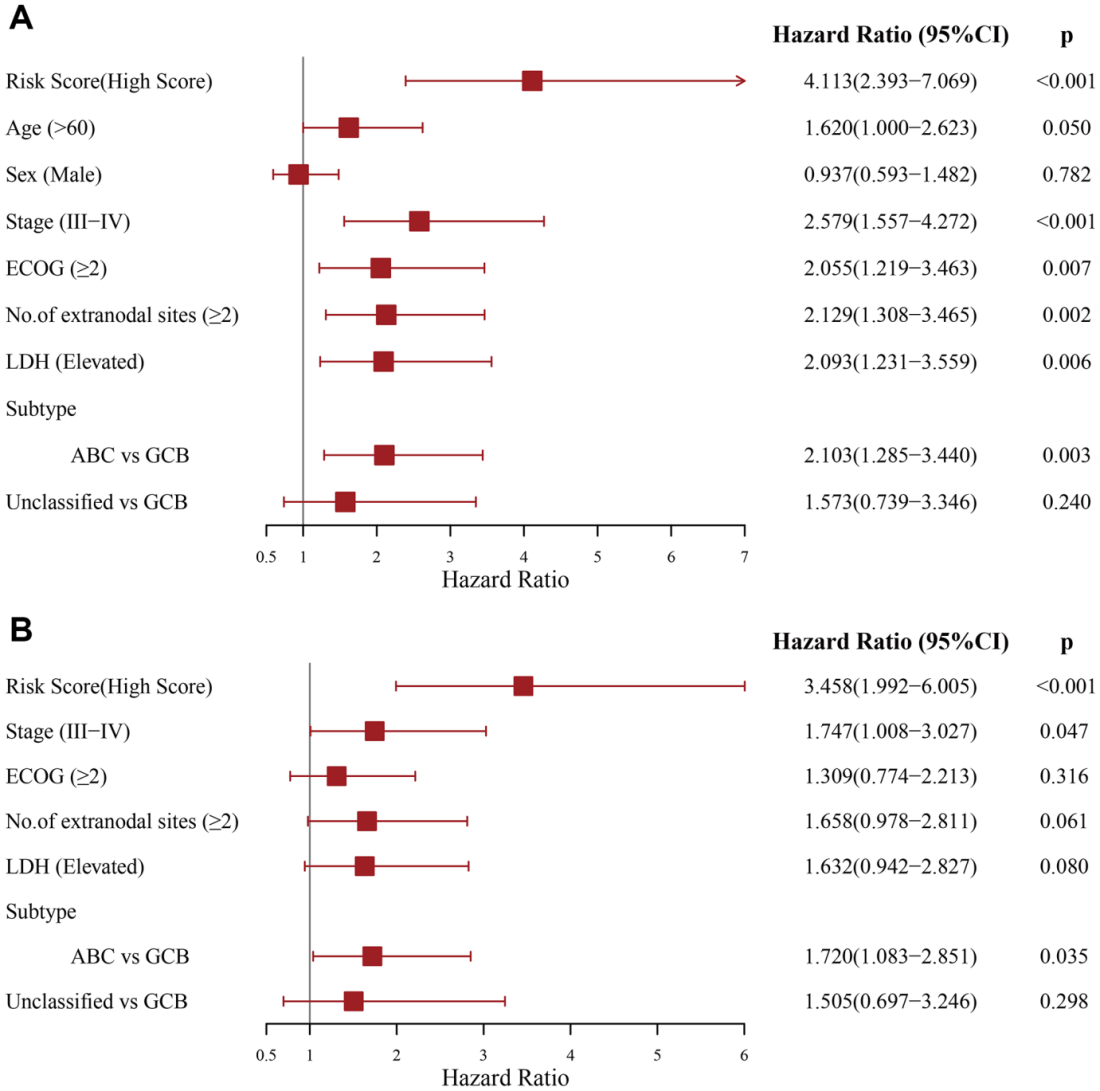
**Evaluation of the independent prognostic value of the prognostic signature in the discovery cohort.** (**A**) Univariate and (**B**) multivariate Cox regression analyses of the signature and clinical factors.

Moreover, the PS correlated significantly with OS in our univariate analysis (HR = 4.113, 95% CI: 2.393–7.069, *p* < 0.001) (Figure 3A). After multivariable adjustment by other clinical factors, including stage, ECOG, LDH, number of sites of extranodal disease and subtype, the PS remained a significant and independent prognostic indicator in the DC (HR = 3.458, 95% CI: 1.992–6.005, *p* < 0.001) (Figure 3B).

### Verification of the PS performance

**Figure 4:**
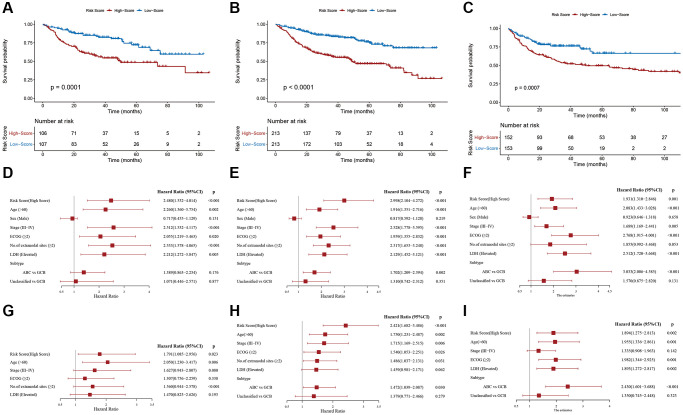
**Validation of the prognostic signature.** Kaplan-Meier plots of overall survival between high- and low-risk patients in the (**A**) internal validation cohort, (**B**) entire GSE31312 cohort, and (**C**) external validation cohort. Univariate Cox regression analyses in the (**D**) internal validation cohort, (**E**) entire GSE31312 cohort, and (**F**) external validation cohort. Multivariate Cox regression analysis in the (**G**) internal validation cohort, (**H**) entire GSE31312 cohort, and (**I**) external validation cohort.

To further assess the robustness of the PS, we performed similar analyses in the IVC, entire GSE31312 cohort (EGC) and external validation cohort (EVC) from the GSE10846 dataset, and all cohorts yielded similar results. Kaplan-Meier survival analysis revealed markedly worse OS in HR patients (Figure 4A–4C). In univariable Cox regression analysis, an elevated RS was an OS risk factor in all validation cohorts (IVC: HR = 2.480, 95% CI: 1.532–4.014, *p* < 0.001 (Figure 4D); EGC: HR = 2.998, 95% CI: 2.104–4.272, *p* < 0.001 (Figure 4E); EVC: HR = 1.931, 95% CI: 1.310–2.846, *p* = 0.001 (Figure 4F)). A similar result was obtained in multivariate Cox regression analysis, in which the PS was analyzed in combination with other clinical factors (Figure 4G–4I). Furthermore, stratification analyses in the EGC indicated worse OS for HR patients in each stratum, including age, sex, stage, ECOG, LDH, number of sites of extranodal disease and subtype, than LR patients, except in the subgroup of those with the unclassified subtype (Supplementary Figure 1). Based on these data, our established PS is a robust and independent predictor of OS in different populations. In addition, HR patients tend to have shorter progression-free survival (PFS) (Supplementary Figure 2). HR patients achieved a remarkably low overall response rate (ORR) and complete remission (CR) rate (ORR, 86.38% vs. 94.84%, *p* = 0.003; CR, 66.20% vs. 84.04%, *p* < 0.001) (Table 1).

**Table 1:** Treatment responses of patients in the entire GSE31312 cohort.

	**Patients in the entire GSE31312 cohort (*n* = 426) nodal-DLBCL**		***p***
	**High-risk group *n* (%)**	**Low-risk group *n* (%)**	
Total number	213 (50)	213 (50)	
ORR	184 (86.38)	202 (94.84)	0.003^*^
CR	141 (66.20)	179 (84.04)	<0.001^**^
PR	43 (20.18)	23 (10.80)	0.007^*^
PD/SD	15/14 (13.62)	6/5 (5.16)	0. 003^*^

Abbreviations: ORR: overall response rate; CR: complete response; PR: partial response; SD: stable disease; PD: progressive disease. ^*^p < 0.05; ^**^p < 0.001.

### Evaluation of the relationship between the PS and clinical factors

**Figure 5:**
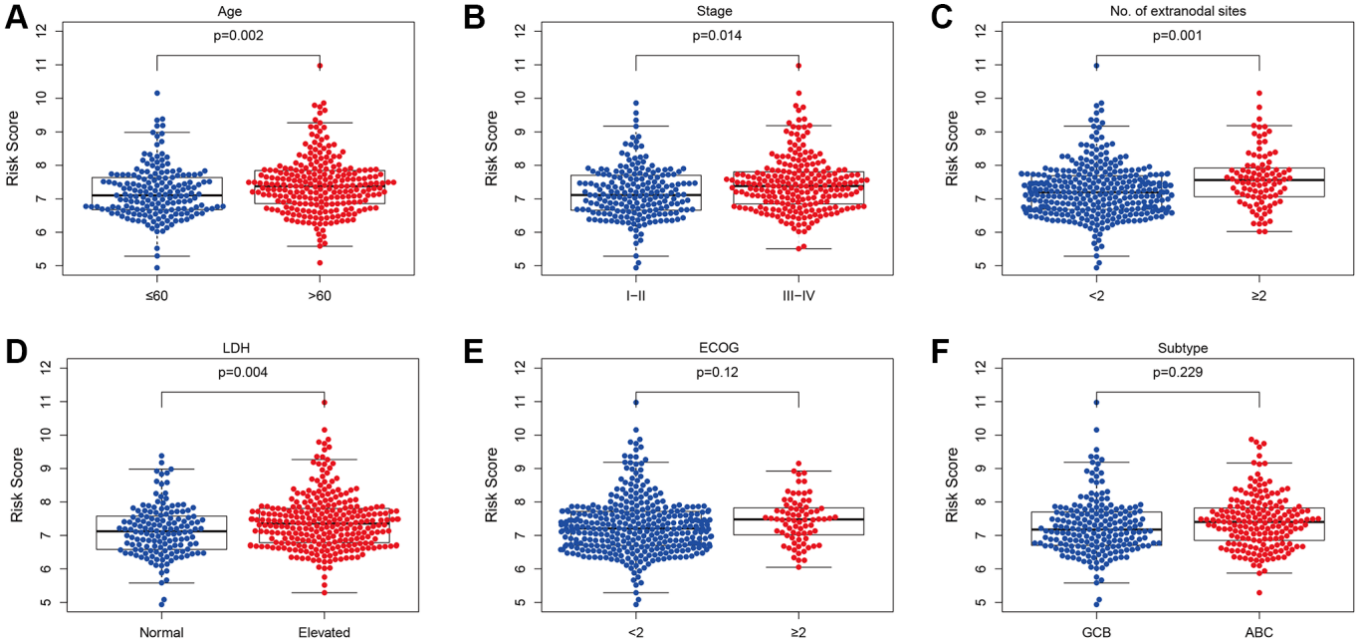
**Relationship between the prognostic signature and clinical factors.** (**A**) Age. (**B**) Stage. (**C**) Number of extranodal sites. (**D**) LDH. (**E**) Subtype. (**F**) ECOG (^*^p < 0.05, ^****^p < 0.0001).

The relationship between the PS and different clinical factors was assessed using the EGC. Compared with patients aged ≤ 60, the RS was increased in patients aged > 60 (*p* = 0.002, Figure 5A). A similar phenomenon was observed for stage (*p* = 0.014, Figure 5B), number of sites of extranodal disease (*p* = 0.001, Figure 5C) and LDH (*p* = 0.004, Figure 5D). However, we did not observe differences in RS regarding ECOG classification (*p* = 0.12, Figure 5E) or subtype classification (*p* = 0.229, Figure 5F).

### Association between the PS and TME

**Figure 6:**
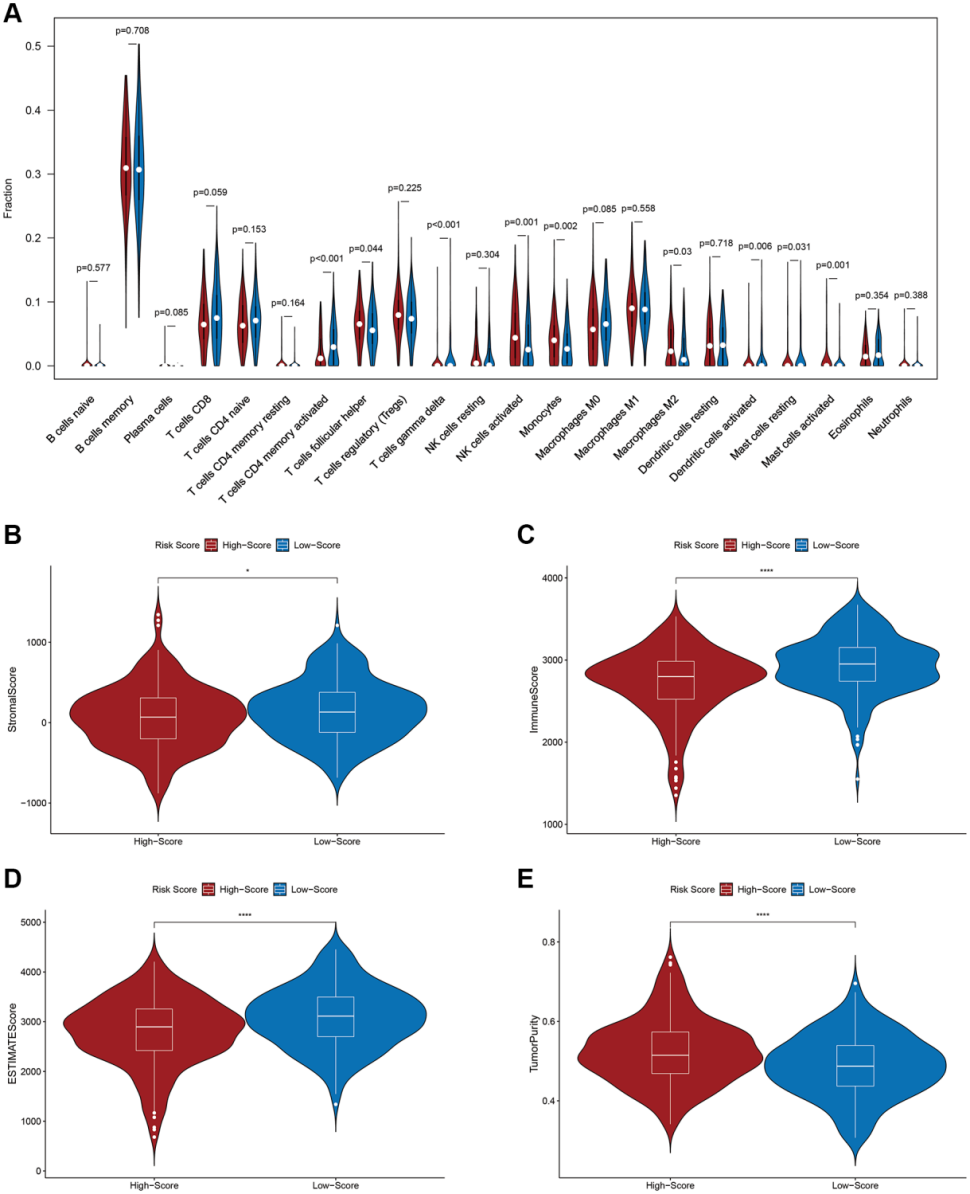
**Relationship between the prognostic signature and the tumor immune microenvironment.** (**A**) Relationship between the prognostic signature and immune cell infiltration. (**B**) Stromal score. (**C**) Immune score. (**D**) ESTIMATE score. (**E**) Tumor purity.

The proportions of follicular helper T cells (*p* = 0.044), activated NK cells (*p* = 0.001), monocytes (*p* = 0.002), M2 macrophages (*p* = 0.030) and activated mast cells (*p* = 0.001) were markedly enhanced in HR patients. Conversely, the numbers of activated memory CD4 T cells (*p* < 0.001), gamma delta T cells (*p* < 0.001), stimulated dendritic cells (*p* = 0.007) and resting mast cells (*p* = 0.031) were significantly decreased in this group (Figure 6A). It was demonstrated that an elevated RS was strongly correlated with tumor purity (*p* < 0.0001) through the ESTIMATE algorithm. However, an elevated RS displayed an inverse correlation with the stromal (*p* < 0.05), immune (*p* < 0.0001) and ESTIMATE scores (*p* < 0.0001) (Figure 6B–6E). Hence, the PS may be reflective of the TME status in DLBCL patients.

### Gene set enrichment analysis (GSEA) for functional annotation of the PS

**Figure 7:**
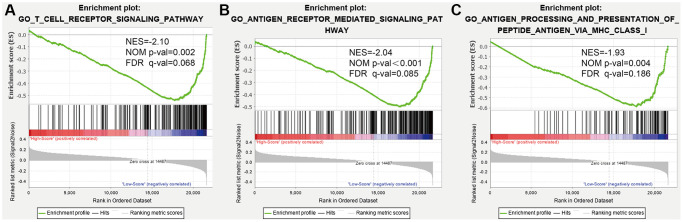
**Gene set enrichment analysis (GSEA) for functional annotation of the prognostic signature.** (**A**) The T cell receptor signaling pathway. (**B**) The antigen receptor-mediated signaling pathway. (**C**) Antigen processing and presentation of peptide antigen via MHC class I.

According to our results, immune-related biological networks were enriched in LR patients, compared to HR patients. We identified three immune-related GO terms in the GSEA results (Figure 7), including T cell receptor, antigen receptor mediated, antigen processing and presentation of peptide antigen via the MHC class I signaling pathway.

## DISCUSSION

Most traditional biomarkers used thus far have weak prognostic power and cannot reflect the status of tumor immune infiltration in DLBCL. Immune-related genes and cells participate throughout the process of malignant tumor initiation, proliferation, and progression [10]. Previous reports indicated that immune-associated long noncoding RNA [11], immune cell constitution [12] and B7-CD28 gene family expression [13] can estimate DLBCL patient prognosis. Here, we developed a novel PS, based on immune-related genes to estimate DLBCL patient prognosis.

Our signature contains four immune-related genes with prognostic power. Among them, CD48 is a protective factor with HR < 1; the three other genes (IL1RL1, PSMD3, RXFP3) are risk factors with HR > 1.CD48 is a member of the signaling lymphocyte activation molecule family that contributes to the activation and proliferation of T cells, antigen presenting cells and granulocytes by binding to CD2 [14]. Wang et al. reported that high CD48 expression activates NK cell function and reverses acute myeloid leukemia immune escape [15]. IL1RL1, an IL-1-type receptor, is detected in a subcategory of T cells and mature myeloid cells [16]. The cytokine IL-33 is the only reported ligand for IL1RL1. The IL-33/IL1RL1 network was shown to contribute to multiple types of blood malignancies [17]. The IL1RL1/IL-33 axis can remodel the tumor stroma or microenvironment to promote malignancy by recruiting a cohort of immune cells [18]. PSMD3 is a member of the 19 S regulatory complex in the 26 S proteasome, participating in cell cycle progression, apoptosis, and DNA damage repair [19]. In acute myeloid leukemia, patients with high level of PSMD3 mRNA have a poor prognosis [20]. PSMD3 promoted NF-κB protein expression and was upregulated in TKI-resistant chronic myeloid leukemia (CML) cells. The level of PSMD3 mRNA was higher in patients with blast phase than in patients with the chronic phase of the disease [21]. RXFP3 belongs to the insulin superfamily [22]. When combined with the ligand, RXFP3 activates downstream kinase pathways via multiple networks, such as, protein kinase C. The *RXFP3* methylation status has a strong correlation with microsatellite instability in endometrial cancer [23]. Overall, the role of these four genes in DLBCL development and immune regulation deserves further investigation.

The signature developed in this study demonstrated excellent predictive performance and effectively classified DLBCL patients into HR and LR categories. In the DC, the HR patients exhibited markedly worse OS, than the LR patients. Furthermore, the immune-related signature is an independent stand-alone prognostic factor, based on multivariate analysis. The results from the validation cohorts agreed with the above results. In addition, the signature showed a strong correlation with clinicopathologic factors. Therefore, this PS may serve as a reliable tool in guiding clinical work.

Immune cells identify tumor cells and destroy them via immune surveillance [24, 25], and immune cell infiltration is a major determinant of DLBCL prognosis [26, 27]. In this study, the proportions of activated memory CD4 T and dendritic cells were lower, but those of M2 macrophages, monocytes and NK cells were significantly higher in HR patients. In general, CD4^+^ T, dendritic, and NK cells are crucial factors in antitumor immunity and have critical significance for cancer immunotherapy [28–30]. Previous studies have reported that high levels of CD4^+^ T cells are associated with improved survival outcomes in many malignancies [31], and mouse models of B–cell lymphoma suggest that CD4^+^ T cells are key to the establishment of an antitumor microenvironment [32]. Dendritic cells have a strong ability to present antigens, and improved antigen presentation has been shown to be a key determinant of survival in patients with DLBCL [33, 34]. Indeed, Ciavarella et al. concluded that DLBCL patients with elevated amounts of dendritic and CD4^+^ T cells experienced prolonged OS. However, patients with a high number of activated NK cells experienced a worse prognosis [35], which is consistent with the results of this study. M2 macrophages are immunosuppressive cells and are thought to be involved in tumor immune evasion [36]. M2 macrophages are a crucial factor for poor survival outcomes and a stand-alone indicator of short OS and PFS [37]. Larger amounts of immune and stromal cells equated to lower quantities of tumor cells [38]. Here, we found that an elevated RS correlated positively with tumor purity but negatively with immune, stromal and ESTIMATE scores. Hence, patients with elevated RSs have more tumor cells and fewer stromal cells. Finally, GSEA showed enrichment of immune-related biological processes in LR patients. Based on this evidence, the TME of patients with low RSs tends to display active immune status and enhanced immune defense. In contrast, that of patients with high-RSs tends to be suppressed. This may explain why the prognosis of HR patients was quite poor.

The advantage of this study is that both internal and external validation were used to evaluate the PS. However, there were several limitations. First, as the data analyzed were downloaded from an online database, our study was retrospective. Second, there are no experimental data to confirm our findings. Thus, more functional studies on these four genes, alone and in combination, are needed to verify the predictive power of the PS and to explore possible immune-related pathways in depth. Our work was the first to report an immune-related gene PS that predicts OS in DLBCL patients.

In conclusion, the signature developed in this study can both predict DLBCL patient survival outcomes and reveal the immunologic status of DLBCL. The PS may be clinically employed to improve patient OS and individualized therapy methods based on RSs. However, both experimental and clinical research efforts are warranted to confirm the findings of this research.

## MATERIALS AND METHODS

### Data source and preprocessing

Transcriptome data (.CEL files) for DLBCL patients were extracted from the GSE31312 and GSE10846 datasets in the Gene Expression Omnibus (GEO) database (https://www.ncbi.nlm.nih.gov/geo/). The raw data were uniformly normalized with the robust multichip average (RMA) technique [39] using the “affy” and “affyPLM” packages, and gene expression profiles were performed on the GPL570 (Affymetrix Human Genome U133 Plus 2.0 Array) platform. For genes with several probes, gene expression values were generated with the median of multiple probes.

After excluding patients without complete clinical information, 426 DLBCL patients (GSE31312 cohort) and 305 DLBCL patients (GSE10846 cohort) were examined in this study; the detailed clinical data are shown in Table 2. Immune-related genes were retrieved from the Immunology Database and Analysis Portal (ImmPort) database (https://www.immport.org) [40].

**Table 2:** Clinical and pathological characteristics of patients with DLBCL in this study.

**Variables**	**GSE31312 N (%)**	**GSE10846 N (%)**
Age, years		
≤60	179 (42.02)	146 (47.87)
>60	247 (57.98)	159 (52.13)
Sex		
Female	183 (42.96)	134 (43.93)
Male	243 (57.04)	171 (56.07)
Stage		
I–II	200 (46.95)	144 (47.21)
III–IV	226 (53.05)	161 (52.79)
No. of extranodal sites		
<2	331 (77.70)	282 (92.46)
≥2	95 (22.30)	23 (7.54)
ECOG		
<2	350 (82.16)	230 (75.41)
≥2	76 (17.84)	75 (24.59)
LDH		
Normal	148 (34.74)	153 (50.16)
Elevated	278 (65.26)	152 (49.87)
Subtype		
GCB	203 (47.65)	133 (43.61)
ABC	183 (42.96)	125 (40.98)
Unclassified	40 (9.39)	47 (15.41)

Abbreviations: ECOG: Eastern Cooperative Oncology Group; LDH: lactate dehydrogenase; GCB: germinal center B-cell-like lymphoma; ABC: activated B-cell-like lymphoma.

### Generation of an immune-related gene PS

In total, 426 samples from the GSE31312 dataset were randomly allocated at a 1:1 ratio to discovery and IVCs using R software. A description and comparison of the baseline features of patients from the DC and IVC was conducted (Supplementary Table 1). Variables were analyzed via the chi-square test, and *p* < 0.05 was the significance threshold.

Univariate analysis was performed to identify immune-related genes strongly related to OS in DC (*p* < 0.001). Next, random forest analysis and least absolute shrinkage and selection operator (LASSO) analysis were conducted simultaneously. The overlapping genes were subjected to multivariate Cox analysis to fulfill variable selection, with *p* < 0.05 as the criterion. We then calculated the scorers, according to a linear combination of the gene levels and regression coefficient of the multivariate Cox analysis: RS = exp_mRNA1_ × β_mRNA1_ + exp_mRNA2_ × β_mRNA2_ … + exp_mRNAi_ × β_mRNAi_, where exp_mRNAi_ is the expression value of each gene, and β_mRNAi_ is the regression coefficient of the multivariate analysis for the candidate gene. The patients were then separated into HR and LR groups, based on the median RS. The Kaplan-Meier log-rank test and time-dependent receiver operating characteristic (ROC) curve analysis were applied to validate PS performance. An area under the ROC curve (AUC) > 0.60 was considered an acceptable prediction value; AUC > 0.75 was regarded as excellent for prediction. RS distribution plots, survival status scatter plots, and expression heatmaps of the four immune-related risk genes between the HR and LR groups were generated to assess PS, and stand-alone prognostic analysis was carried out to evaluate whether this signature was indeed an independent stand-alone predictor of OS.

### Application and verification of the immune-related gene PS

RSs for each patient in the IVC, the EGC and the EVC from the GSE10846 dataset were computed and assigned to two groups, according to the median. Kaplan-Meier log-rank tests and univariate and multivariate analyses were conducted to compare OS between the HR and LR groups. Kaplan-Meier log-rank tests were performed to compare PFS between the HR and LR groups in the DC, IVC and EGC. The treatment response in the EGC was analyzed by Pearson’s chi-square test.

### Evaluation of the relationship between the PS and clinical factors

To assess the predictive value of PS in DLBCL, we examined the correlation between PS and clinical factors in the EGC. Intergroup differences were analyzed by independent Student’s *t*-tests. Two-tailed *p* < 0.05 was set as the significance threshold.

### Association between the PS and TME

The proportion of 22 immune cell subtypes based on expression profile data in the EGC was assessed using the CIBERSORT package [41], with permutations set at 1000. Cases with *p* < 0.05 according to the CIBERSORT results were included in further analyses. The Wilcoxon test was employed to compare differences in immune cell subtypes between the HR and LR groups. To further evaluate the association between this signature and the TME, the stromal, immune, and tumor purity scores were computed using the ESTIMATE algorithm [42]. Significance was considered at *p* < 0.05.

### GSEA

GSEA [43] was performed to examine different biological processes between the HR and LR patients. The gene expression profiles of the EGC were evaluated with regard to Gene Ontology (GO) gene sets. The number of random sample permutations was set at 1000, and enriched gene sets with a nominal *p* < 0.05 and FDR < 0.25 were regarded as significant.

### Statistical analysis

In this study, all statistical analyses were carried out with R version 3.6.3 (https://www.r-project.org/) and the corresponding packages.

## Supplementary Materials

Supplementary Figures

Supplementary Table 1
