# Supplementary material for: A novel prognostic signature based on immune-related genes of diffuse large B-cell lymphoma
Source: Aging (Albany NY). 2021 Oct 5;13(19):22947–62. doi: 10.18632/aging.203587 (PMC8544299; doi:10.18632/aging.203587)
Supplement: Supplementary Figures [file aging-13-203587-s001.pdf]

SUPPLEMENTARY FIGURES

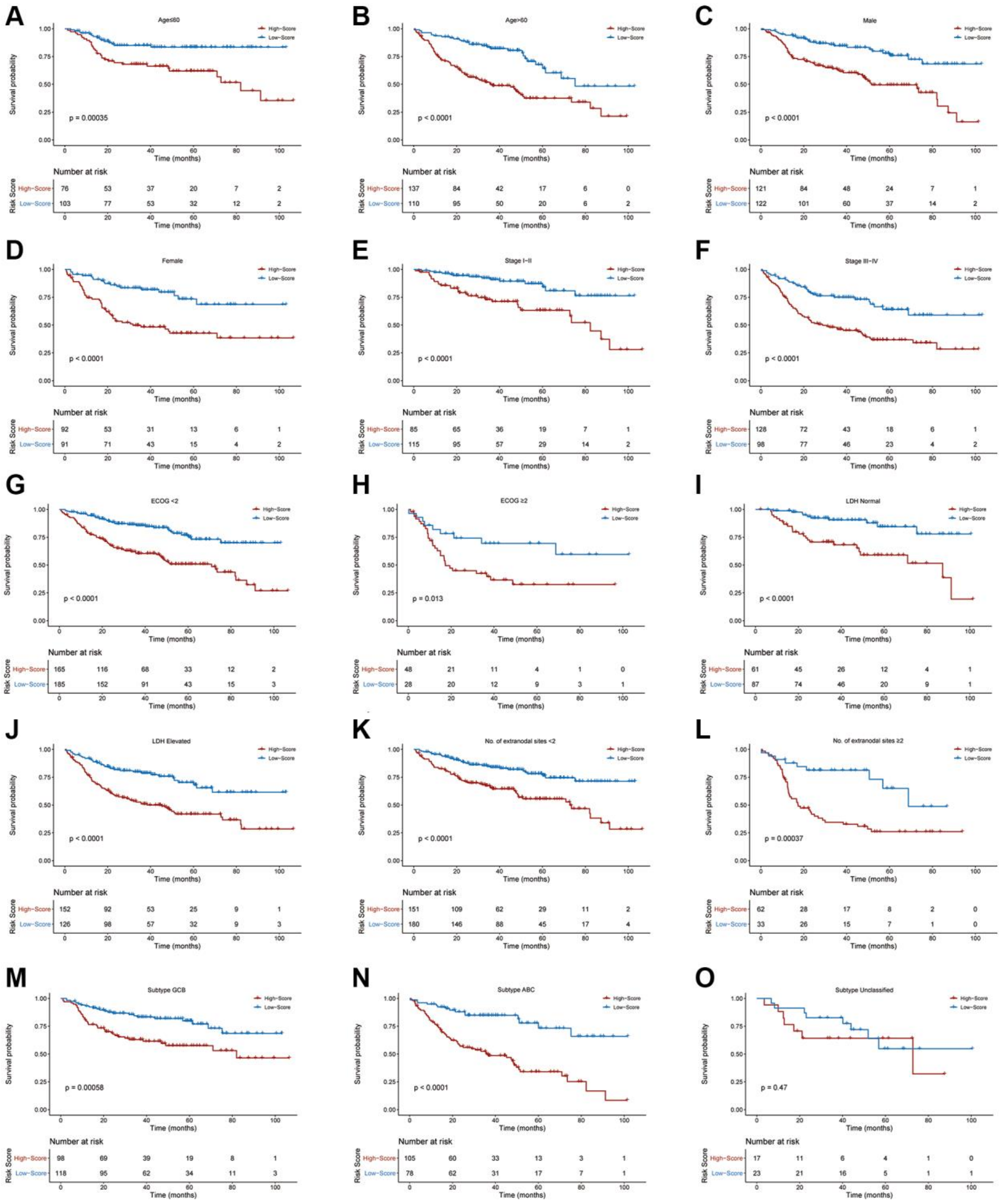

Supplementary Figure 1. Stratification analyses of overall survival between high- and low-risk patients in different subgroups. (**A**) Age ≤ 60. (**B**) Age > 60. (**C**) Male. (**D**) Female. (**E**) Stage I–II. (**F**) Stage III–IV. (**G**) ECOG < 2. (**H**) ECOG ≥ 2. (**I**) LDH Normal. (**J**) LDH Elevated. (**K**) Number of extranodal sites < 2. (**L**) Number of extranodal sites ≥ 2. (**M**) Subtype GCB. (**N**) Subtype ABC. (**O**) Subtype Unclassified.

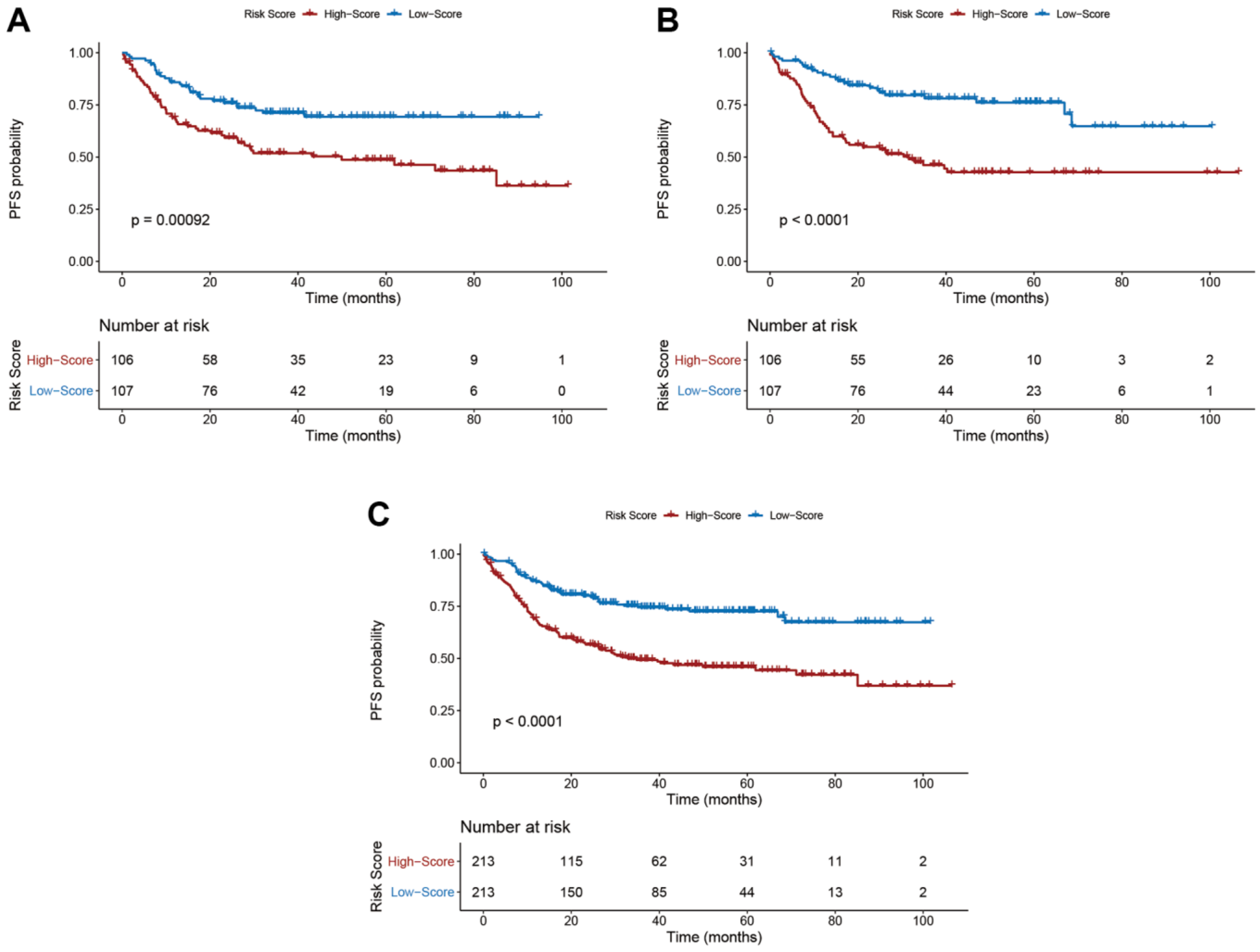

Supplementary Figure 2. Kaplan-Meier plots of progression-free survival between high- and low-risk patients. (**A**) Discovery cohort, (**B**) internal validation cohort, (**C**) entire GSE31312 cohort.
