# Supplementary material for: A novel prognostic signature based on immune-related genes of diffuse large B-cell lymphoma
Source: Aging (Albany NY). 2021 Oct 5;13(19):22947–62. doi: 10.18632/aging.203587 (PMC8544299; doi:10.18632/aging.203587)
Supplement: Supplementary Table 1 [file aging-13-203587-s002.pdf]

SUPPLEMENTARY TABLE

Supplementary Table 1. Clinical and pathological characteristics of patients with DLBCL in discovery and internal validation cohort.

Variables	GSE31312 N (%)	GSE10846 N (%)
Age, years		
≤60	179 (42.02)	146 (47.87)
>60	247 (57.98)	159 (52.13)
Sex		
Female	183 (42.96)	134 (43.93)
Male	243 (57.04)	171 (56.07)
Stage		
I–II	200 (46.95)	144 (47.21)
III–IV	226 (53.05)	161 (52.79)
No. of extranodal sites		
<2	331 (77.70)	282 (92.46)
≥2	95 (22.30)	23 (7.54)
ECOG		
<2	350 (82.16)	230 (75.41)
≥2	76 (17.84)	75 (24.59)
LDH		
Normal	148 (34.74)	153 (50.16)
Elevated	278 (65.26)	152 (49.87)
Subtype		
GCB	203 (47.65)	133 (43.61)
ABC	183 (42.96)	125 (40.98)
Unclassified	40 (9.39)	47 (15.41)

Abbreviations: ECOG: Eastern Cooperative Oncology Group; LDH: lactate dehydrogenase; GCB: germinal center B-cell-like lymphoma; ABC: activated B-cell-like lymphoma. Variables were checked via χ^2-test.
